# Glutamate delta-1 receptor regulates cocaine-induced plasticity in the nucleus accumbens

**DOI:** 10.1038/s41398-018-0273-9

**Published:** 2018-10-12

**Authors:** Jinxu Liu, Pauravi J. Gandhi, Ratnamala Pavuluri, Gajanan P. Shelkar, Shashank M. Dravid

**Affiliations:** Department of Pharmacology, Creighton University School of Medicine, Omaha, NE 68178 USA

## Abstract

Cocaine exposure induces plasticity of glutamatergic synapses of medium spiny neurons (MSNs) in the nucleus accumbens (NAc), which has been proposed to contribute to its addictive behavior. The mechanisms underlying cocaine-induced plasticity are not fully understood. The orphan glutamate delta-1 (GluD1) receptor is a member of the ionotropic glutamate receptor family but does not function as a typical ligand-gated ion channel. Instead it serves a synaptogenic function by interacting with presynaptic Neurexin protein. Recent neuroanatomical studies have demonstrated enriched expression of GluD1 in the NAc but its role in reward behavior, MSN function, and drug-induced plasticity remains unknown. Using a combination of constitutive and conditional GluD1 KO models, we evaluated the effect of GluD1 ablation on cocaine-conditioned place preference (CPP) and cocaine-induced structural and functional plasticity. GluD1 KO mice showed higher cocaine CPP. Selective ablation of GluD1 from striatal neurons but not cortico-limbic excitatory neurons reproduced higher CPP. Higher cocaine preference in GluD1 KO correlated with an increase in spine density, greater maturation of dendritic spines, and basally upregulated spine-regulating active cofilin. GluD1 loss did not affect basal excitatory neurotransmission or plasticity but masked the generation of cocaine-induced silent synapses. Finally, loss of GluD1 increased the GluN2B subunit contribution to NMDA receptor currents in MSNs and a partial agonist of GluN2B-containing NMDA receptors normalized the higher active cofilin and cocaine preference in GluD1 KO mice. Together, these findings demonstrate a critical role of GluD1 in controlling susceptibility to cocaine preference and cocaine-induced plasticity by modulating NMDA receptor subunit contribution.

## Introduction

Cocaine-induced neuroplasticity in the nucleus accumbens (NAc) has been proposed to contribute to its addictive behavior. Plasticity in dendritic spines of medium spiny neurons (MSNs) and glutamatergic neurotransmission is observed after cocaine exposure^1–8^. Several molecular mediators of spine plasticity have been identified including the Rac1-cofilin pathway^9^. An increase in the active form of cofilin is observed upon cocaine exposure and constitutively active cofilin and dominant negative Rac1 increase cocaine preference^9^. Functionally, cocaine exposure has been found to induce generation of silent synapses in MSNs^5,10^ and impairing maturation of silent synapses reverses incubation of cocaine craving^11,12^. Generation of silent synapses, which contain NMDA receptors but lack AMPA receptors, correlates with an increase in the expression of GluN2B subunit^10^. The role of several intracellular PSD components has been evaluated to account for these functional changes in synapses^13–15^; however, the role of synaptogenic factors, such as adhesion molecules, is still lacking.

The glutamate delta-1 (GluD1) is a member of the delta family of ionotropic glutamate receptors but does not exhibit typical ligand-induced ion channel currents^16,17^. Instead the glutamate delta receptors are endowed with the ability to form and maintain synapses by forming a *trans*-synaptic bridge involving presynaptic partner Neurexin and the mediator molecule Cbln1^18,19^. In addition, GluD1 is critical for regulation of postsynaptic signaling^20^. Recently, we have demonstrated that GluD1 modulates dendritic spine-regulating cofilin signaling. GluD1 deletion increases levels of active cofilin in cortico-limbic region and affects spine dynamics^21^. Loss of GluD1 also leads to an impaired developmental switch in NMDA receptor subunits and GluD1 KO have higher GluN2B to GluN2A subunit expression ratio. Furthermore, in agreement with the genetic association of *GRID1* gene that codes for GluD1 with autism^22^ and schizoaffective disorders^23,24^, we have found that GluD1 KO mice exhibit social deficits, repetitive behavior, depression-like behavior, and hyperaggression^21,25,26^. Together, the molecular phenotypes upon loss of GluD1 and the association of GluD1 with neuropsychiatric disorders, which have comorbidites with substance abuse^27,28^, may have implications for cocaine-induced behavioral and plasticity effects. Importantly, recent neuroanatomical studies have identified enriched expression of GluD1 in NAc^29,30^ and GluD1 has been identified to play a role in the regulation of dopaminergic neurons that send inputs to NAc^31^.

In this study, we evaluated the role of GluD1 in cocaine-induced plasticity in the NAc. We found that GluD1 KO mice have higher preference for cocaine in CPP test and exhibit significantly more cocaine-induced structural plasticity and spine maturation compared to wildtype. No change in basal excitatory neurotransmission and mGluR1/5-induced plasticity was observed in GluD1 KO, but cocaine-induced generation of silent synapses was masked in GluD1 KO mice. We also found MSNs in GluD1 KO mice have a higher GluN2B-NMDAR component. Finally, pharmacological normalization of NMDA receptor subunit contribution using d-cycloserine, a partial agonist for GluN2B-containing receptors, normalized upregulated active cofilin, and higher cocaine preference in GluD1 KO mice. Together, these results demonstrate a critical role of GluD1 in the regulation of cocaine preference and cocaine-induced plasticity by modulating neurotransmission and signaling at glutamatergic synapses.

## Methods and materials

### Animals

Male wildtype and GluD1 KO mice^32^ were used for these studies. Mice were group housed at a constant temperature (22 ± 1 °C) and a 12-h light–dark cycle with free access to food and water as previously described^26^. GluD1^flox/flox^ mice were obtained from Dr. Pei Lung-Chen with loxP sites in intron 10 and 12. The GluD1^flox/flox^ mice were crossed with Rgs9-cre^33^ and Emx1-cre^34^ driver mice to selectively ablated GluD1 from the striatum and excitatory neurons, respectively. Studies were conducted in accordance with the recommendations in the Guide for Care and Use of Laboratory Animals of the National Institutes of Health. All experimental protocols were approved by the Creighton University Institutional Animal Care and Use Committee Policies and Procedures.

### Immunohistochemistry

Immunohistochemistry was performed for GluD1 or p-cofilin using methodology as previously described^21,35^. Additional details are included in supplementary methods.

### Behavior

For cocaine-conditioned place preference (CPP) test mice were conditioned to associate with two compartments of the choice apparatus; one compartment with drug injection and another with saline injection. Behavioral testing was performed between 9:00 a.m and 4:00 p.m. in mice 8–10 weeks of age. After habituation, pre-test was conducted to detect baseline preference for the two compartments; black (smooth floor, translucent top) versus white (serrated floor, transparent top). Conditioning was conducted one day after pre-test. Cocaine was injected on one side (black or white) while saline was injected in the other compartment. Two sessions (cocaine–saline) were conducted each day for 3 days and thereafter post-test was performed. The CPP sessions were video-recorded and scored using Any-maze for time spent in the two compartments. The change in time spent (Δ time) in the cocaine-associated chamber from pre-test to post-test, referred to as preference, was analyzed. For testing the effect of d-cycloserine (Sigma, Saint Louis, MO) on CPP, mice received one intraperitoneal injection of d-cycloserine (320 mg/kg) one day prior to the beginning of first conditioning session.

### Dendritic spine analysis

Diolistic analysis of MSN dendritic spine density and morphology was performed using method as previously described^21,35^. Additional details are included in supplementary methods.

### Slice electrophysiology

Whole-cell patch clamp recordings were performed as previously described^21,35^. Additional details are included in supplementary methods.

### Statistics

Sample sizes were chosen based on our previous studies with GluD1 KO mice. Most experiments were replicated in three independent groups. Data were analyzed using Student’s unpaired *t*-test, one-way ANOVA, or two-way ANOVA with post-hoc multiple comparisons test. Differences were considered significant if *P* < 0.05. Prism 6 (GraphPad Software Inc., San Diego, CA, USA) was used for analysis and representation.

## Results

### Deletion of GluD1 from striatum increases cocaine preference

**Fig. 1:**
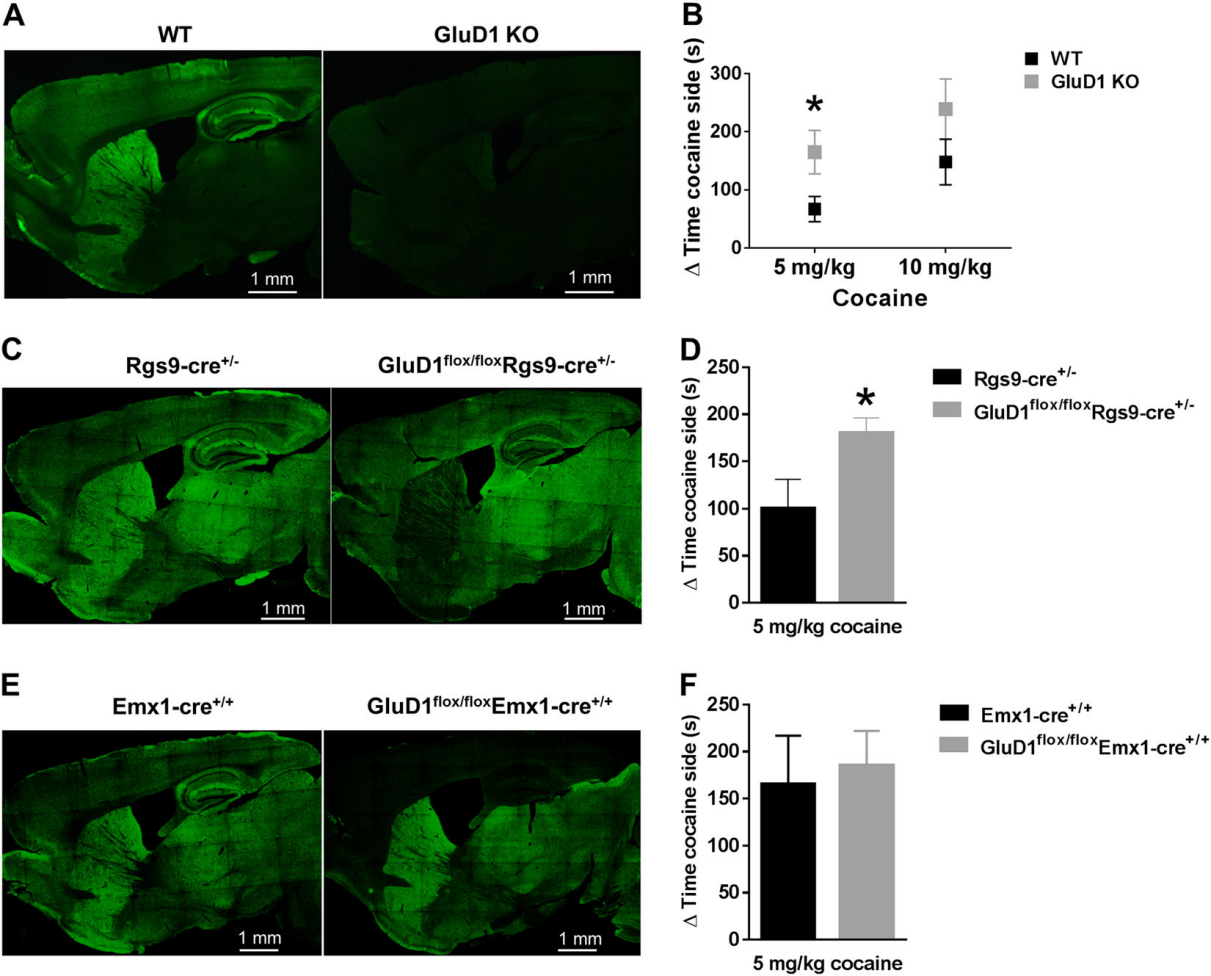
Striatal GluD1 loss enhances cocaine preference. **a** Expression of GluD1 in mouse brain was assessed using immunohistochemistry. No labeling was observed in GluD1 KO confirming antibody specificity. **b** CPP testing was conducted which included three conditioning days. The change in time spent in the cocaine-associated chamber between pre-test and post-test was determined. A significantly higher time spent was observed in GluD1 KO at 5 mg/kg dose (*p = 0.033, *n* = 8 WT, 5 GluD1 KO). A trend for higher preference in GluD1 KO was observed at a dose of 10 mg/kg (*n* = 9 each for WT and GluD1 KO). **c** Selective ablation of GluD1 from striatum was achieved by using a Rgs9-Cre driver line. Immunohistochemistry demonstrated lack of GluD1 expression specifically in the striatum. **d** Ablation of striatal GluD1 led to facilitation of cocaine preference (*p = 0.024, *n* = 6 WT, 10 striatal KO). **e** Using a Emx1-Cre driver line, GluD1 was conditionally deleted from the excitatory neurons in cortico-limbic regions. **f** No significant effect on cocaine CPP was observed upon deletion of GluD1 from excitatory neurons (*n* = 6 WT, 7 cortico-limbic KO)

We first evaluated the effect of GluD1 deletion on cocaine preference using CPP test. In preliminary studies we established the dose for these experiments. We found that 5 mg/kg dose represented a subthreshold dose whereas 10 and 15 mg/kg led to maximal effect on cocaine preference. Thus, we tested the effect of 5 and 10 mg/kg dose on CPP in wildtype and GluD1 KO mice. We found that GluD1 KO mice spent significantly more time on the cocaine (5 mg/kg) injected side compared to wildtype (WT 67.13 ± 21.86 s vs. GluD1 KO 165.2 ± 37.48 s, *p* *=* 0.033, unpaired *t*-test). A trend was also observed for higher preference at 10 mg/kg dose of cocaine in GluD1 KO but this was not significant (Fig. 1b). In order to address the specific brain nuclei underlying GluD1 control of cocaine preference we utilized a conditional knockout strategy. We used a Rgs9-cre driver line together with the GluD1^flox/flox^ line to ablate GluD1 from the striatum. In this model reduced expression of GluD1 was observed in the striatum but not in other brain regions (Fig. 1c). As seen in Fig. 1d, GluD1^flox/flox^Rgs9-cre^+/−^ mice showed higher preference for cocaine compared to Rgs9-cre^+/−^ mice (Rgs9-cre^+/−^ mice 100.4 ± 30.4 s vs. GluD1^flox/flox^Rgs9-cre^+/−^ mice 180.3 ± 15.95 s, *p* *=* 0.024, unpaired *t*-test). We also tested whether ablation of GluD1 from cortico-limbic excitatory neurons using the Emx1-Cre driver produce changes in cocaine preference (Fig. 1e). As seen in Fig. 1f, no difference in cocaine preference was observed upon GluD1 ablation from excitatory neurons (*p* *=* 0.75, unpaired *t*-test). Together, the use of conditional approach suggests a critical role of striatal GluD1 in cocaine preference.

### Cocaine induces greater dendritic spine plasticity in GluD1 knockout mice

**Fig. 2:**
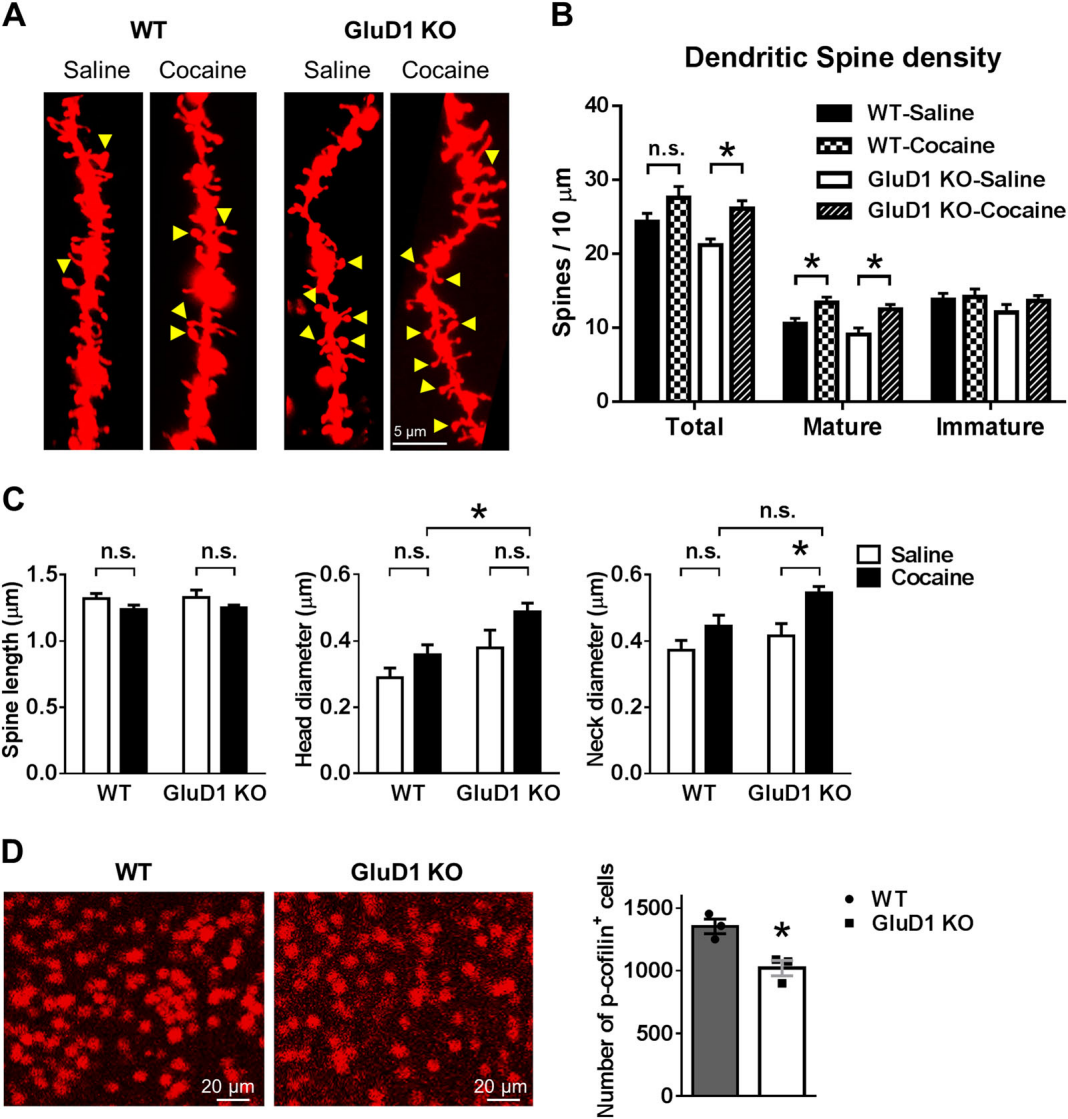
Facilitated cocaine-induced spine plasticity and maturation in GluD1 KO. **a** Representative images of dendritic spines analyzed by disolistic labeling and 3-D reconstruction after cocaine (5 mg/kg) CPP in wildtype and GluD1 KO. Arrowheads indicate spines with head and neck. Scale bar = 5 µm. **b** Total dendritic spine density was increased in cocaine-treated GluD1 KO *p = 0.045; *n* = 21 (WT saline), 20 (WT cocaine), 15 (KO saline), 27 (KO cocaine) dendrites/3 mice/group; two-way ANOVA with Tukey’s post-hoc test; cocaine treatment, *F*_1,76_ = 10.37, *p* = 0.0019; genotype, *F*_1,76_ = 3.24, *p* = 0.076; interaction, *F*_1,76_ = 0.45, *p* = 0.5). Mature dendritic spine density was also increased in cocaine-treated WT (*p = 0.041) and GluD1 KO (*p = 0.019; two-way ANOVA with Tukey’s post-hoc test; cocaine treatment, *F*_1,76_ = 16.29, *p* = 0.0001; genotype, *F*_1,76_ = 2.22, *p* = 0.14; interaction, *F*_1,76_ = 0.12, *p* = 0.73). **c** Mean spine length, head diameter, and neck diameter was calculated for each dendrite and effect of genotype and drug was evaluated. Dendritic spines in GluD1 KO exhibited greater maturation upon cocaine treatment as evident by a significant increase in neck diameter (*p = 0.016; two-way ANOVA with Tukey’s post-hoc test; cocaine treatment, *F*_1,79_ = 11.76, *p* = 0.001; genotype, *F*_1,79_ = 5.92, *p* = 0.017; interaction, *F*_1,79_ = 0.91, *p* = 0.34). The head diameter of cocaine-treated GluD1 KO was significantly higher than cocaine-treated wildtype (*p = 0.023; two-way ANOVA with Tukey’s post-hoc test; cocaine treatment, *F*_1,79_ = 7.06, *p* = 0.0096; genotype, *F*_1,79_ = 10.49, *p* = 0.0018; interaction, *F*_1,79_ = 0.35, *p* = 0.55). **d** Lower number of inactive p-cofilin-labeled neurons was observed in GluD1 KO (*p *=* 0.017, unpaired *t*-test, *n* = 3 mice/genotype)

Cocaine exposure is known to induce plastic changes in dendritic spines of NAc MSNs which correlate with its addictive behavior. To address the role of GluD1 in structural plasticity we conducted cocaine CPP as described above, and thereafter collected the tissue for diolistic labeling and dendritic spine analysis. We observed a significant increase in total spine density in NAc core MSNs in GluD1 KO but not in wildtype mice exposed to cocaine compared to saline controls (two-way ANOVA, WT saline 24.306 ± 1.192 vs. cocaine 27.545 ± 1.562, *p* = 0.25; GluD1 KO saline 21.168 ± 0.847 vs. cocaine 26.112 ± 1.075, *p* = 0.045) (Fig. 2b). An increase in mature type spines was observed in both wildtype and GluD1 KO (two-way ANOVA, WT saline 10.511 ± 0.764 vs. cocaine 13.377 ± 0.768, *p* = 0.041; GluD1 KO saline 9.094 ± 0.901 vs. cocaine 12.486 ± 0.654, *p* = 0.019). Furthermore, we found that the dendritic spines in cocaine-conditioned GluD1 KO mice underwent greater maturation with a significant increase in neck diameter (two-way ANOVA, WT saline 0.372 ± 0.03 µm vs. cocaine 0.445 ± 0.033 µm, *p* = 0.29; GluD1 KO saline 0.416 ± 0.037 µm vs. cocaine 0.545 ± 0.02 µm, *p* = 0.016) (Fig. 2c). In addition, the head diameter was significantly higher in GluD1 KO cocaine vs. WT cocaine (*p* = 0.023, two-way ANOVA). Cocaine-induced spine plasticity is controlled by Rac1-cofilin signaling^9^. Thus, we tested whether there is a change in active cofilin levels in GluD1 KO. We found lower number of the inactive p-cofilin-labeled MSNs in GluD1 KO suggesting that the actin-severing pathway may be more active in GluD1 KO (WT 1355 ± 58.77 vs. GluD1 KO 1022 ± 60.64, *p* = 0.017, unpaired *t*-test) (Fig. 2d). Together, these results suggest that GluD1 KO are more sensitive to cocaine-induced structural plasticity which may underlie higher cocaine preference.

### **Loss of GluD1 does not affect basal excitatory neurotransmission andmGluR1/5long-term depression (LTD) in the NAc**

**Fig. 3:**
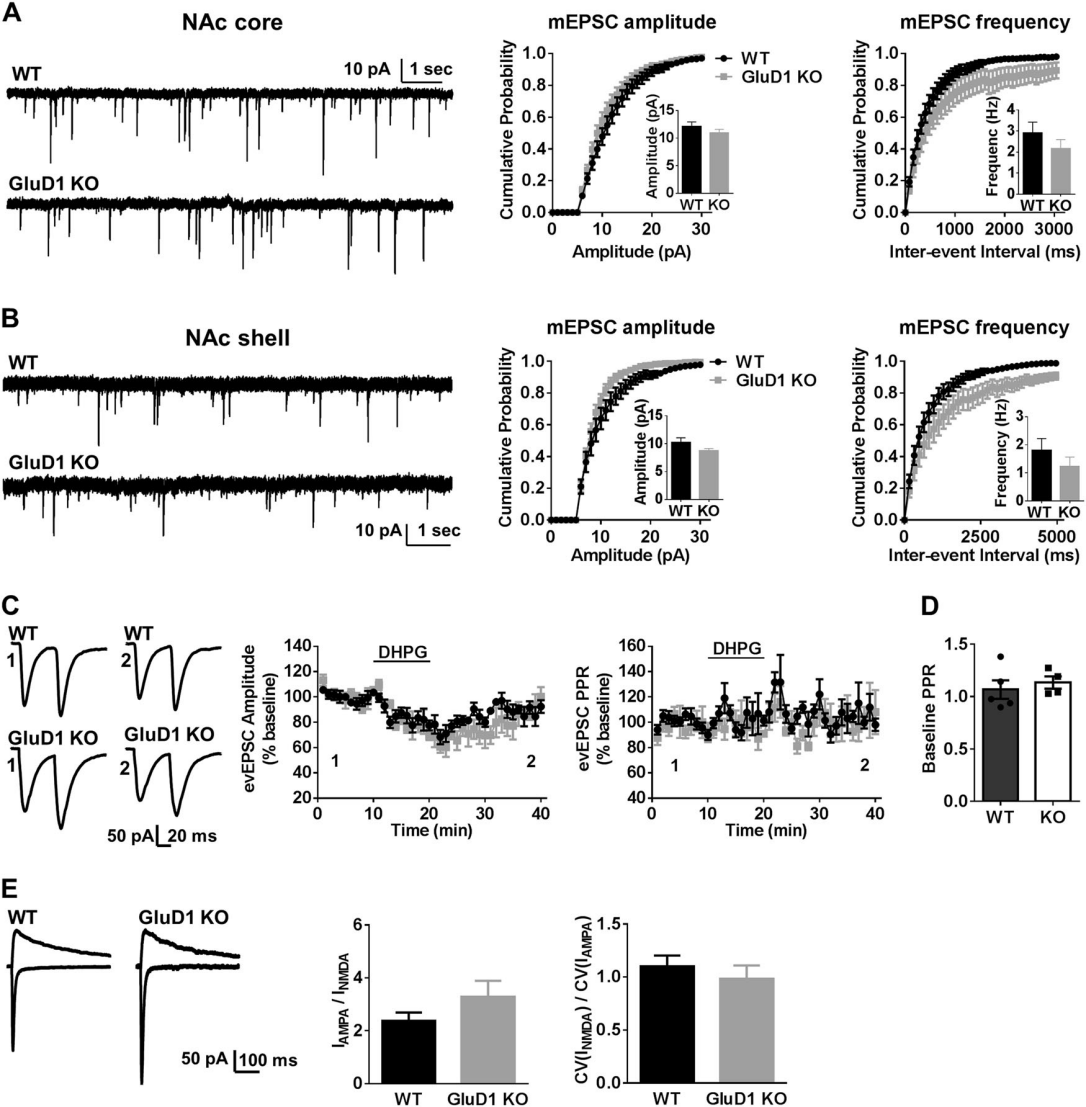
GluD1 loss does not affect basal excitatory neurotransmission and plasticity in NAc. **a** Whole-cell voltage-clamp recordings from MSNs were conducted. No significant change in mEPSC amplitude or frequency was noted in NAc core (*n* = 11 (WT), 13 (KO) neurons). **b** No significant change in mEPSC characteristics was noted in NAc shell (*n* = 10 neurons). **c** Chemical mGluR1/5 LTD was addressed at corticostriatal synapses in MSNs. DHPG-induced depression of evoked AMPA receptor responses was assessed in WT and GluD1 KO. No significant change in LTD was observed (*n* = 5 (WT), 4 (KO) neurons). **d** No change in paired-pulse facilitation was observed in GluD1 KO. **e** The ratio of AMPA/NMDA receptor currents or the ratio of coefficient of variance of AMPA responses to NMDA responses, a representative of silent synapses, was not different in GluD1 KO (*n* = 10 (WT), 9 (KO) neurons)

We next evaluated whether there are changes in excitatory neurotransmission due to loss of GluD1. We first evaluated miniature excitatory postsynaptic currents (mEPSC) in wildtype and GluD1 KO. We found that there were no significant changes in the amplitude (core, *p* = 0.27; shell, *p* = 0.14; unpaired *t*-test) or frequency (core, *p* = 0.28; shell, *p* = 0.29; unpaired *t*-test) of mEPSC in NAc core or shell (Fig. 3a, b) in GluD1 KO. We have recently shown that GluD1 KO mice exhibit changes in mGluR1/5 signaling^20^, a pathway associated with reward behavior^36,37^. Thus, we further tested whether mGluR1/5 agonist DHPG-induced LTD was affected in the MSNs. Bath application of DHPG produced a significant reduction in the amplitude of evoked EPSCs. However, no difference in DHPG-LTD was observed between WT and GluD1 KO (*p* = 0.43, unpaired *t*-test) (Fig. 3c). In addition no change in paired-pulse ratio of evoked EPSCs at corticostriatal synapses was observed (*p* = 0.57, unpaired *t*-test) (Fig. 3d). We did not observe any significant deficit in either AMPA/NMDA ratio (*p* = 0.19, unpaired *t*-test) or coefficient of variation (CV) ratio (a measure of silent synapses) in GuD1 KO (*p* = 0.47, unpaired *t*-test) (Fig. 3e). Together, there is no change in basal neurotransmission and mGluR1/5 plasticity in MSNs in NAc of GluD1 KO mice.

### GluD1 deletion masks cocaine-induced functional plasticity of glutamatergic synapses

**Fig. 4:**
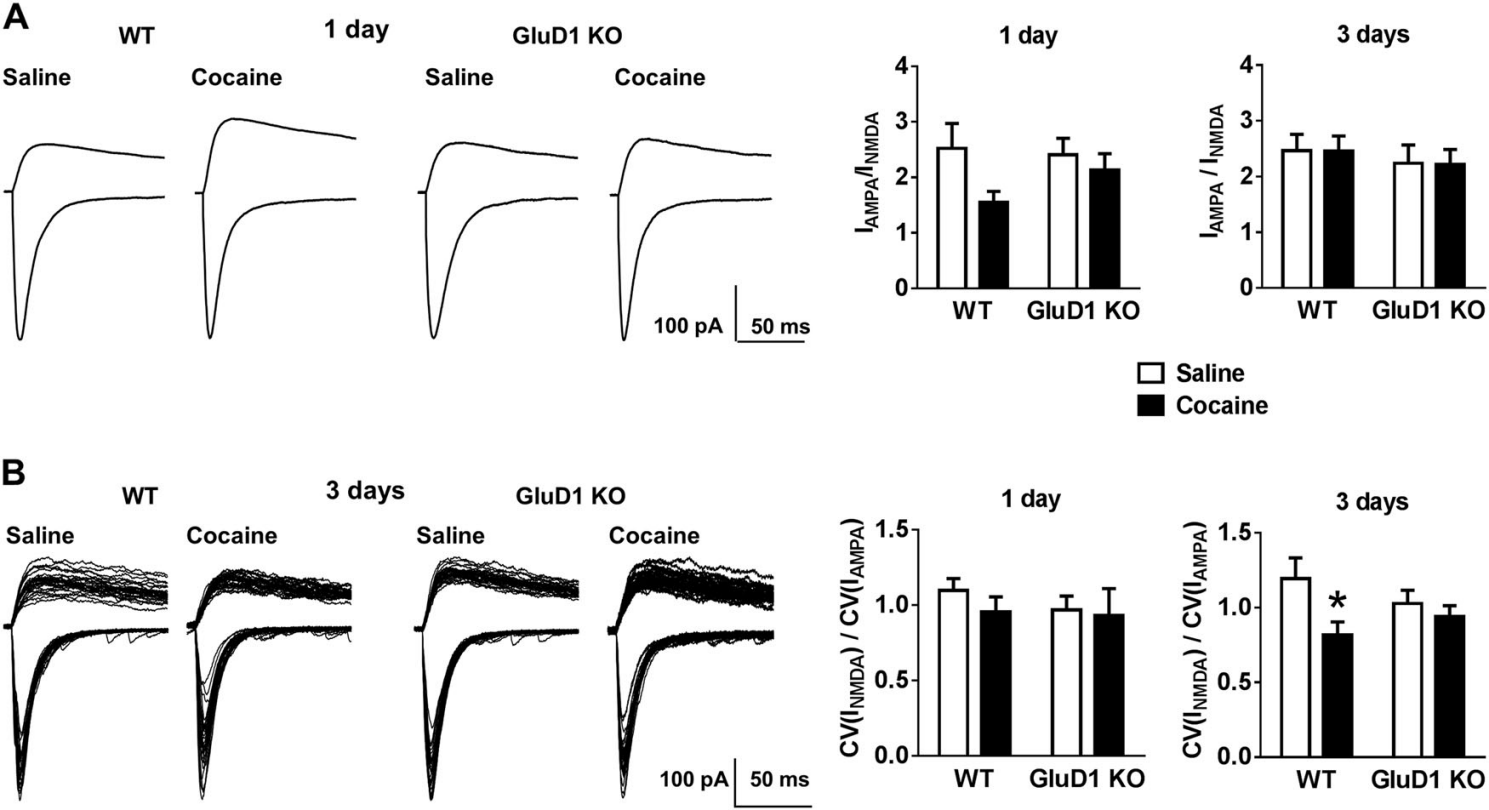
Cocaine-induced generation of silent synapses was absent in GluD1 KO. **a** Mice were injected with cocaine (5 mg/kg) for 1 day or for 3 consecutive days and the effect on AMPA/NMDA ratio was observed 24 h later. No drug or genotype effect was observed [*n* = 1 day; 10 (WT sal), 9 (WT cocaine), 9 (KO sal), 8 (KO cocaine); 3 days 11 (WT sal), 13 (WT cocaine), 10 (KO sal), 12 (KO cocaine)]. **b** Wildtype mice that received repeated cocaine injection demonstrated an increase in silent synapses as evidenced by a reduction in the ratio of CV of AMPA receptor responses to NMDA receptor responses (*p = 0.042; two-way ANOVA with Tukey’s post-hoc test; cocaine treatment, *F*_1,42_ = 5.54, *p* = 0.023; genotype, *F*_1,42_ = 0.049, *p* = 0.83; interaction, *F*_1,42_ = 2.11, *p* = 0.15)

Cocaine exposure induces changes in expression and function of glutamate receptors at MSN synapses and leads to generation of silent synapses^6,8,10^. We tested the effect of cocaine at a dose (5 mg/kg) that led to higher preference in GluD1 KO on changes in AMPA/NMDA ratio and occurrence of silent synapses. We first assessed changes one day after a single injection of cocaine (5 mg/kg i.p.). Although no significant difference in the AMPA/NMDA ratio was observed after one-day cocaine treatment in GluD1 KO (WT saline vs. WT cocaine, *p* = 0.17; GluD1 KO saline vs. GluD1 KO cocaine, *p* = 0.99; two-way ANOVA with Tukey’s post-hoc test) (Fig. 4a), a trend for lower AMPA/NMDA ratio was observed in cocaine-treated wildtype mice compared to saline (*p* = 0.07, unpaired *t*-test). Furthermore, the occurrence of silent synapse was measured by analyzing the ratio of the CV of the I_NMDA_ to the CV of the I_AMPA_, an increase in silent synapses would be detected as a decrease in this ratio^5^. One-day cocaine exposure did not produce a change in ratio of CV in wildtype or GluD1 KO suggesting lack of silent synapse generation (WT saline vs. WT cocaine, *p* = 0.78; GluD1 KO saline vs. GluD1 KO cocaine, *p* = 0.99; two-way ANOVA with Tukey’s post-hoc test) (Fig. 4b). We then injected cocaine for 3 consecutive days (5 mg/kg, i.p.) and tested synaptic plasticity one day after the last injection. No change in the synaptic strength AMPA/NMDA ratio was observed under these conditions. Interestingly, 3-day cocaine treatment increased the occurrence of silent synapses in wildtype but not in GluD1 KO (two-way ANOVA, WT saline 1.194 ± 0.1384 vs. cocaine 0.8173 ± 0.08865, *p* = 0.042; GluD1 KO saline 1.028 ± 0.08871 vs. cocaine 0.9394 ± 0.07352, *p* = 0.92) (Fig. 4b). Thus, repeated cocaine administration-induced generation of silent synapses was absent in GluD1 KO.

### GluD1 KO have higher contribution of GluN2B subunit to NMDA receptor currents in MSNs that accounts for higher cocaine preference

**Fig. 5:**
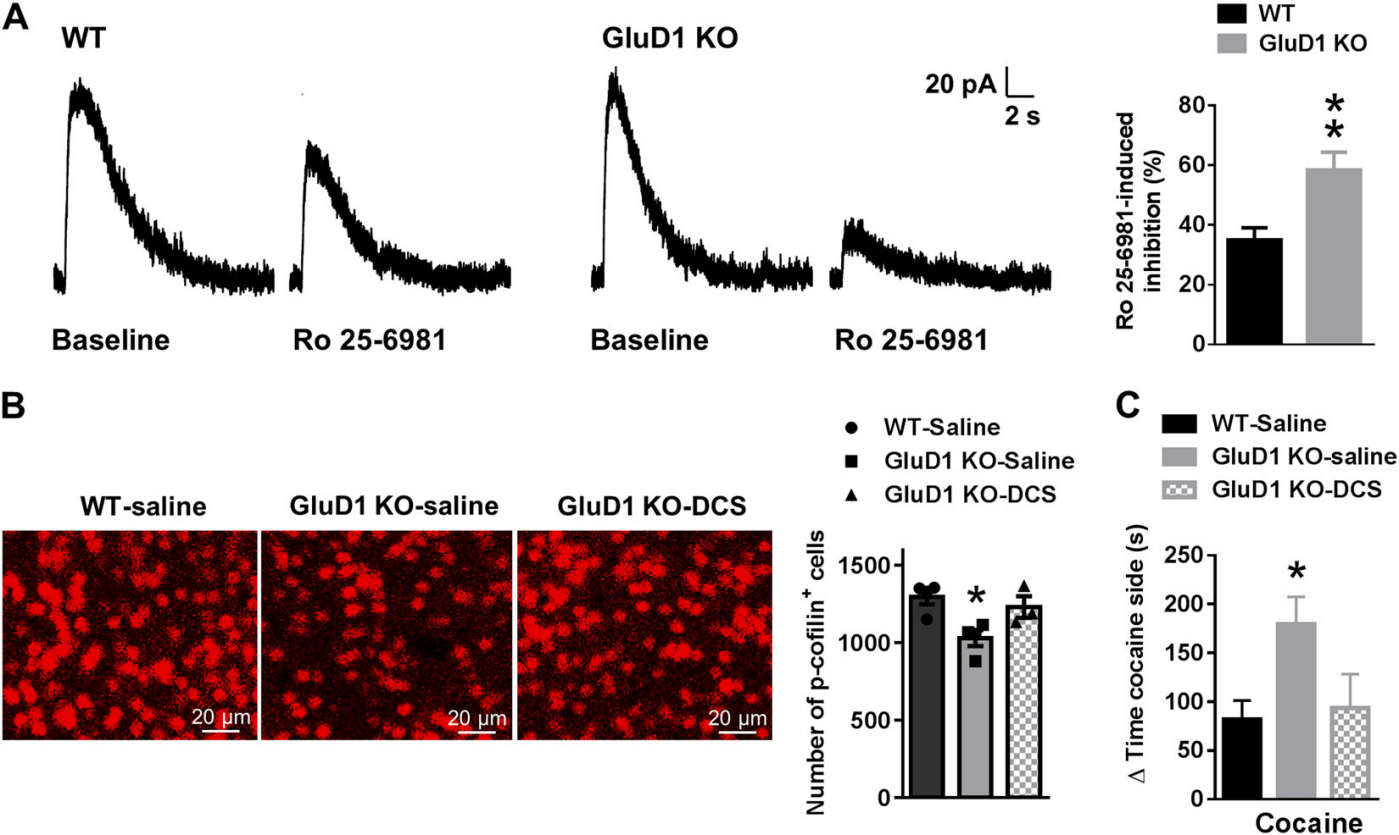
Higher GluN2B subunit component in NMDA receptor currents in GluD1 KO underlies cocaine preference **a** NMDA receptor responses were induced by puff of 1 mM glutamate and the effect of bath applied GluN2B antagonist Ro-25-6981 (1 µM) was evaluated. A higher GluN2B component was observed in GluD1 KO (**p = 0.008; *n* = 6 neurons from three mice per genotype). **b** The effect of d-cycloserine (320 mg/kg) a partial agonist at GluN2B-containing receptors was evaluated on basal p-cofilin levels. Levels of p-cofilin were evaluated one day after saline or d-cycloserine administration. d-cycloserine normalized the lower p-cofilin levels in GluD1 KO (*p = 0.018; one-way ANOVA with Tukey’s post-hoc test; between groups, *F*_2,8_ = 6.78, *p* = 0.019; *n* = 4 (WT sal), 4 (KO sal), 3 (KO DCS) mice). **c** d-cycloserine administration one day prior to conditioning reduced the higher cocaine CPP in GluD1 KO (*p = 0.041; one-way ANOVA with Tukey’s post-hoc test; between groups, *F*_2,18_ = 3.86, *p* = 0.04; *n* = 9 (WT sal), 6 (KO sal), 6 (KO DCS) mice)

Silent synapses are synapses that express NMDA receptors and lack AMPA receptors and are therefore inactive under basal condition. Insertion of GluN2B in response to cocaine exposure has been found to lead to formation of silent synapses^5,10,38^. We have previously shown that there is an impairment in the developmental switch in NMDAR subunit in GluD1 KO in the hippocampus and cortex such that GluD1 KO have higher relative expression of GluN2B compared to GluN2A^21^. Shift in NMDA receptor subunit expression may explain the differences in functional plasticity induced by cocaine in GluD1 KO (Fig. 4). Thus, we tested whether GluD1 KO MSNs have differences in GluN2B contribution to NMDA receptor currents. Whole-cell recordings were obtained from NAc neurons and NMDA currents were induced by application of NMDA using picospritzer. Effect of bath application of GluN2B-selective blocker Ro-25-6981(1 µM) was evaluated. We found that Ro-25-6981 inhibited NMDA-induced currents and the degree of inhibition was greater in GluD1 KO compared to wildtype demonstrating greater contribution of GluN2B subunit in GluD1 KO (WT 35.03 ± 4.004% inhibition vs. GluD1 KO 58.39 ± 5.901% inhibition, *p* = 0.0083, unpaired *t*-test) (Fig. 5a). Interestingly, NMDA-induced responses were also significantly higher in GluD1 KO (WT 102.84 ± 16.40 pA vs. GluD1 KO 197.49 ± 16.86 pA, *p* < 0.001). Because at 1 µM concentration Ro-25-6981 primarily inhibits diheteromeric GluN1/GluN2B receptors and has low efficacy for triheteromeric GluN1/GluN2A/GluN2B^39,40^, similar to other GluN2B-selective inhibitors^41,42^, it may be concluded that there may be an increase in diheteromeric GluN1/GluN2B receptors in GluD1 KO potentially without reduction in GluN1/GluN2A/GluN2B triheteromeric receptors. Also, because we did not observe a significant change in AMPA/NMDA ratio and amplitude of AMPA mEPSC in GluD1 KO, it is possible that the increased GluN2B component may exist extrasynaptically. These conclusions however have limitations because in picospitzer experiments the precise concentration of the agonizts at receptors and the area of neuron that is exposed to agonizts is uncertain.

We further tested whether altered contribution of NMDA receptor subtypes may be responsible for some of the molecular changes and behavioral responses to cocaine in GluD1 KO. We tested the effect of d-cycloserine (320 mg/kg), which is a GluN1 subunit ligand but has partial agonist activity at GluN2B-containing receptors^43^, and may therefore normalize the altered GluN2B subunit contribution in GluD1 KO. We have recently used this dose of d-cycloserine to replicate effect of selective GluN2B inhibitor for molecular and behavioral deficits in GluD1 KO^21^. P-cofilin levels were evaluated one day after intraperitoneal administration of d-cycloserine. We found that d-cycloserine normalized the basally lower levels of p-cofilin (one-way ANOVA, WT saline 1296 ± 48.85 vs. GluD1 KO saline 1030 ± 51.24, *p* = 0.018; GluD1 KO saline 1030 ± 51.24 vs. GluD1 KO DCS 1230 ± 70.11, *p* = 0.088) (Fig. 5b). d-cycloserine administered one day prior to beginning of cocaine conditioning also normalized the higher cocaine CPP in GluD1 KO to saline-wildtype levels (one-way ANOVA, WT saline 82.09 ± 19.29 s vs. GluD1 KO saline 179.3 ± 28.24 s, *p* = 0.041; GluD1 KO saline 179.3 ± 28.24 s vs. GluD1 KO DCS 93.68 ± 34.71 s, *p* = 0.11) (Fig. 5c). Together, these results demonstrate that altered NMDA receptor subunit contribution underlies higher susceptibility to cocaine preference in GluD1 KO.

## Discussion

Our studies have identified a unique role of GluD1 subunit in regulating cocaine preference and cocaine-induced plasticity. Deletion of GluD1 led to an increase in cocaine preference which correlated with an increase in spine density and greater maturation of spines (Figs. 1 and 2). Using a conditional approach, we demonstrated that striatal GluD1 underlie the increase in cocaine preference (Fig. 1b, c). The cofilin signaling pathway may facilitate cocaine-plasticity and cocaine preference in GluD1 KO (Fig. 2), since this pathway has been shown to trigger higher cocaine preference^9^. We also found higher contribution of the GluN2B subunit to the NMDA component in GluD1 KO and a GluN2B partial agonist normalized the basally upregulated active cofilin and cocaine preference (Fig. 5).

### GluD1 deletion generates synapses primed for cocaine plasticity

We evaluated the changes in spine dynamics as a result of cocaine conditioning. We found that in wildtype mice there was a trend for an increase in total spine density and a significant increase in mature spines (Fig. 2). This is in contrast to the increase in thin spines after cocaine treatment that convert to a mature phenotype during abstinence^37,44,45^ and is likely due to the lower dose of cocaine and the conditioning protocol in our studies. A significant increase in both total spine density and mature spines was observed in GluD1 KO (Fig. 2). Thus, the synaptogenic function of GluD1 is not necessary for cocaine-induced increase in dendritic spine density. Furthermore, spines in cocaine treated GluD1 KO attained a more mature phenotype with higher head and neck diameter (Fig. 2). Together with our previous report it is likely that higher GluN2B-mediated cofilin-signaling may underlie this facilitated maturation of spines in GluD1 KO^21^. This is consistent with findings that constitutively active cofilin increases cocaine preference^9^. We hypothesize that spines in GluD1 KO are in a primed-state for maturation upon cocaine exposure. This hypothesis is also consistent with the lack of cocaine-induced generation of silent synapse, a form of immature synapse, in GluD1 KO. Repeated cocaine administration increases the number of silent synapses^5^, which is non-contingent and primarily dependent on pharmacological effect^15^. Cocaine-induced generation of silent synapses involves synaptic insertion of new, GluN2B-containing NMDARs^5^. During abstinence the silent synapses can either be eliminated or stabilized^45^. Because impairing maturation of silent synapses reverses incubation of cocaine craving, the stabilization of silent synapses is considered a mechanism for consolidating cocaine memory^11,12^. We found that repeated administration of low-dose of cocaine in wildtype mice-induced generation of silent synapses (Fig. 4). In GluD1 KO, spine density increase occurred in the absence of generation of silent synapses suggesting increase in synapses with mature phenotype (Fig. 2). Because GluDs govern AMPA receptor trafficking^20,46,47^, it is possible that loss of GluD1 may modulate the normal AMPA receptor trafficking and impair generation of silent synapses. Further molecular studies are necessary to fully understand the role of GluD1 in drug-induced generation of silent synapses.

### Altered NMDA receptor subunit composition underlying neuropsychiatric phenotypes and increased drug preference

Switch in NMDA receptor subunit from enriched GluN2B to GluN2A represents a critical developmental step at glutamatergic synapses^48–51^. Overexpression of GluN2A reduces the number and volume of synapses and inhibits spine dynamics. In contrast, GluN2B overexpression does not affect the total number of synapses but increases spine motility and spine turnover. In addition, GluN2B C-terminal interaction are important for maturation of synapses^52,53^. Thus, the facilitated maturation and robust spine dynamics and active cofilin in GluD1 KO correlate with the higher GluN2B content (Fig. 5). We have previously found that the switch in NMDA receptor subunits is delayed in GluD1 KO leading to higher GluN2B to GluN2A expression ratio in cortex and hippocampus of GluD1 KO^21^. GluN2B selective inhibitor and partial agonist of GluN2B-containing receptors reversed the abnormality in cofilin signaling, spine density, as well as several behavioral phenotypes including depression-like behavior, repetitive behavior and social deficit in GluD1 KO^21,26^. Here, we demonstrate that greater GluN2B subunit contribution may also underlie higher cocaine preference in GluD1 KO (Fig. 5). Together, we propose that developmental delay in NMDA receptor subunit switch and an increase in GluN2B-mediated NMDA receptor current may increase the susceptibility for both neuropsychiatric disorders and addictive behavior, and this may serve as a mechanism for comorbidities among some forms of mental disorders and substance abuse. The mechanism by which GluD1 may regulate NMDA receptor expression and composition is unknown, but it is possible that GluD1-mediated synapse organization is a critical step in the maturation of synapses including the switch in NMDA receptor subunits. Future studies are necessary to address this aspect of GluD1 function.

### Function of GluD1 subunit in the NAc

The GluD1 subunit despite being a member of ionotropic glutamate receptor family does not exhibit typical ligand-gated ionic influx but instead serves a non-traditional synaptogenic function^16,18,19,54^. We found no significant change in mEPSC frequency or amplitude or paired-pulse facilitation suggesting that GluD1 loss does not affect the number of functional synapses in the NAc. Similarly, spine density in saline-treated GluD1 KO was not different from saline-treated wildtype (Fig. 2). Together, these finding suggests that the synaptogenic function of GluD1 is not obligatory in NAc and/or homeostatic mechanisms mediated by other synaptogenic adhesion molecules may compensate for loss of GluD1. This finding is also in agreement with our previous reports in cortico-limbic region^21^. We also found that GluD1 ablation does not affect mGluR1/5-LTD or AMPA/NMDA ratio and basal silent synapse number in the NAc (Fig. 3). In contrast, in the hippocampal CA1 region chemical DHPG-LTD was impaired^20^. However, due to the differences in methodology to detect AMPA receptor-mediated depression (surface biotinylation versus electrophysiology) it is possible that the effect on trafficking of extrasynaptic and synaptic receptors may underlie these differences. Overall our results demonstrate that although loss of GluD1 may not have an effect on the basal neurotransmission in the NAc, more subtle difference, such as change in the contribution of NMDA receptor subunit and overall NMDA receptor currents may affect drug-induced plasticity and behavior. Furthermore, together with our previous report similar mechanisms may also underlie emergence of neuropsychiatric behaviors.

## Electronic supplementary material

Supplementary extended methods
