# Supplementary material for: Glutamate delta-1 receptor regulates cocaine-induced plasticity in the nucleus accumbens
Source: Transl Psychiatry. 2018 Oct 12;8:219. doi: 10.1038/s41398-018-0273-9 (PMC6185950; doi:10.1038/s41398-018-0273-9)
Supplement: Supplementary file 1 — Supplementary extended methods [file 41398_2018_273_MOESM1_ESM.docx]

**Supplementary:**

**Extended Methods:**

*Immunohistochemistry*

Mice were transcardially perfused with 4% PFA in 0.1 M phosphate buffer (PB) and brain were collected and stored overnight in the same fixative at 4 °C. Brains were then transferred successively into solutions of 10%, 20% and 30% sucrose in 0.1 M PB before freezing in isopentane at − 30 °C to − 40 °C. For immunohistochemistry 20 μM thick sagittal or coronal sections were cut using a cryostat (Leica CM 1900, Buffalo Grove, IL). After washing thrice for 5 mins each with 0.1 M PB, sections were incubated in blocking solution containing 10% normal goat serum in 0.25% Triton-X in 0.1 M PB (PBT) for 1 hr at room temperature. Following blocking, sections were incubated overnight at 4 °C in guinea pig anti-GluD1 primary antibody (1:500, GluD1C-GP-Af860, Frontier Institute Co., Ltd, Japan) and rabbit anti-p-cofilin (Ser3) primary antibody (1:20, #3313, Cell Signaling Technology) in PBT. The following day, sections were washed 6 times for 5 mins each in 0.1 M PBT and incubated with the goat anti-guinea pig secondary antibody conjugated to AlexaFluor 488 (1:1000, A-11073, Life Technologies, Eugene, OR) or goat anti-rabbit secondary antibody conjugated to AlexaFluor 594 (1:500, A-11012, Life Technologies) for 2 hours at room temperature in dark. Sections were then washed 6 times for 5 mins each in 0.1 M PBT, mounted on pre-cleaned glass slides and coverslipped with Fluoromount-G (SouthernBiotech, Birmingham, AL). Images were acquired with an Infinity camera (Lumenera Co., Ottawa, ON, Canada) coupled to a fluorescence microscope (Nikon Eclipse Ci, Melville, NY) using the Lumenera Infinity Analyze software (Lumenera Co.) or Olympus VS120 virtual slide scanning systems. P-cofilin positive cells were counted by an Image-Pro Plus 6.0 software (Media cybernetics, Rockville, MD), 4-8 images were analyzed per animal.

*Diolistic labeling and spine analysis*

Diolistic labeling was performed as previously described (Gupta *et al,* 2015; Gupta *et al,* 2016) with minor modifications. The animals were anesthetized and perfused with 0.1 M phosphate buffer (PB) followed by 1.5% paraformaldehyde (PFA). Brains were removed and postfixed in the same fixative for 1 hr before coronal slices of 150 μm thickness were prepared. Tungsten particles (1.3 μm diameter) (Bio-Rad Laboratories, Hercules, California, USA) coated with the lipophilic carbocyanine dye DiI (Molecular Probes, Life Technologies, Grand Island, NY, USA) were delivered diolistically into the section at 120 psi using a Helios Gene Gun system (Bio-Rad Laboratories, Hercules, California, USA) with a modified chamber. A polycarbonate filter with 2.0 μm pore size was capped on top of the gun barrel to filter larger particles. DiI was allowed to diffuse along neuron dendrites and axons in PB containing 0.01% (w/v) thimerosal (Sigma–Aldrich, St. Louis, MO, USA) for 24–48 h at 4 °C, and then labeled sections were fixed again in 4% PFA for 1 h. After brief wash in PB, sections were mounted onto glass slides in Fluoromount-G mounting medium.

Imaging of labeled sections was performed using LSM 510 confocal microscope (Zeiss, Chester, VA, USA). DiI was excited using the Helium/Neon at 543 nm laser line. The entire profile of each DiI-positive neuron to be quantified was acquired using a 63x oil immersion objective. After the neuron was scanned and confirmed as NAc MSN, its dendrites were focused using a 63x oil-immersion objective with 2x magnification and scanned at 0.1 μm intervals along the z-axis. The filament module of Imaris software 8.4.1, a 3-D imaging software (Bitplane, South Windsor, CT, USA) was used to quantify the spine density, and morphology. The minimum end segment diameter (spine head) was set at ≥0.20 μm. Automatic quantification of dendritic protrusions and spine classification into mature and immature was performed using Imaris XT. For quantitative analysis, image was rendered by the surpass module of Imaris software. Spine quantification commenced on dendrites beginning at 75 μm distal to the soma, and secondary dendrites were preferentially sampled. The minimum length of dendrite quantified was 25 μm. Counts were made by rotating the segment in 3-D to be able to quantify spines in the z-axis. For each neuron, one or two dendrites were analyzed. For each group, 5-10 neurons per animal were analyzed and averaged and a total of 3 mice were used for each group. Briefly, parameters for morphological classification of dendritic spines were as follows; mature: length (spine) < 3 μm and maximum width (head) > mean width (neck) × 2 or length < 1 μm; immature: length (spine) > 1 μm and mean width (head) ≥ mean width (neck) or length >3 μm lacking distinct head.

*Slice electrophysiology*

Whole-cell electrophysiology was performed as previously described (Gupta *et al,* 2015; Gupta *et al,* 2016). After isoflurane anesthesia, mice (P28-35) were decapitated and brains were removed rapidly and placed in ice-cold artificial cerebrospinal fluid (ACSF) of the following composition (in mM): 130 NaCl, 24 NaHCO3, 3.5 KCl, 1.25 NaH2PO4, 2.4 CaCl2, 2.5 MgCl2 and 10 glucose saturated with 95% O2/5% CO2. 300 μm thick parasagittal sections were prepared using vibrating microtome (Leica VT1200, Buffalo Grove, IL, USA). Whole-cell patch recordings were obtained from core and shell of nucleus accumbens medium spiny neurons in voltage-clamp configuration with an Axopatch 200B (Molecular Devices, Sunnyvale, CA, USA). Glass pipette with a resistance of 3-5 mOhm were filled with an internal solution consisting of (in mM) 126 cesium methanesulfonate, 8 NaCl, 10 HEPES, 8 Na2-phosphocreatine, 0.3 Na2GTP, 4 MgATP, 0.1 CaCl2, 1 EGTA (pH 7.3). 2.9 mM QX-314 was added to block voltage-gated sodium channels. Signal was filtered at 2 kHz and digitized at10 kHz using an Axon Digidata 1440A analog-to-digital board (Molecular Devices, CA). Whole-cell recordings with a pipette access resistance less than 20 mOhm and that changed less than 20% during the duration of recording were included.

EPSCs were evoked by a bipolar tungsten electrode (World Precision Instruments, FL, USA) placed at the border between NAc core and the cortex dorsal to the anterior commissure. To determine AMPA receptor-to-NMDA receptor ratio, peak amplitude of EPSCs at -70 mV in presence of 100 µM picrotoxin was measured as AMPA receptor-mediated currents and peak amplitude of EPSCs at +40 mV in presence of 100 µM picrotoxin and 10 µM CNQX was measured as NMDA receptor-mediated currents. 30 consecutive trials were recorded at 0.1 Hz for each condition. Amplitudes of AMPA receptor- and NMDA receptor-EPSC were calculated by averaging 30 EPSCs at each condition. The coefficient of variation (CV) was obtained by dividing the square root of the variance with the EPSC amplitude mean. Long term depression (LTD) induced by bath application of 100 µM R, S-DHPG was monitored while evoking 50 ms paired pulses at 0.05 Hz.

Neurons were held at a holding potential of -70 mV for mEPSCs. For recording mEPSCs 0.5 µM tetrodotoxin and 100 µM picrotoxin were added to the external recording solution. The mEPSC recordings were analyzed using Minianalysis software (Synaposoft, Atlanta, GA, USA) with an amplitude threshold set at 5 pA. Frequency and amplitude of the miniature currents were determined.

For Ro 25-6981experiments, a picospritzer (Parker Hannifin) was used to evoke currents by pressure applying brief pulses (7-9 psi; 10-50 ms) of 1 mM glutamate through a borosilicate capillary tube (1-2 mOhm). During the picospritzer recordings, the neurons were hold at +40 mV with the recording solution containing 0.5 µM tetrodotoxin, 100 µM picrotoxin and 10 µM CNQX. Five stable baseline measurements were obtained at 20 s interval followed by bath application of 1 µM Ro 25-6981 for 15 min. The last 5 measurements in the presence of Ro 25-6981 were used for analysis. Finally, The NMDA receptor competitive antagonist D,L-AP5 (100 µM) was applied which completely inhibited the currents (data not shown).
